# The four serotypes of dengue recognize the same putative receptors in *Aedes aegypti *midgut and *Ae. albopictus *cells

**DOI:** 10.1186/1471-2180-6-85

**Published:** 2006-10-02

**Authors:** Ricardo F Mercado-Curiel, Héctor Armando Esquinca-Avilés, Rosalinda Tovar, Álvaro Díaz-Badillo, Minerva Camacho-Nuez, María de Lourdes Muñoz

**Affiliations:** 1 Department of Genetics and Molecular Biology, Centro de Investigación y de Estudios Avanzados del Instituto Politécnico Nacional. Ave. Instituto Politécnico Nacional 2508 Col San Pedro Zacatenco, C.P. 07360, México, D. F., México; 2 Centro de Investigación en Ciencia Aplicada y Tecnología Avanzada del Instituto Politécnico Nacional, Postgrado en Tecnología Avanzada, Legaria 694, Col. Irrigación, Delg. Miguel Hidalgo C.P. 11500 México D. F., México; 3 Genomic Sciences Program, Universidad Autónoma de la Ciudad de México, Fray Servando Teresa de Mier, # 99, Col. Centro, CP. 06080, México D. F., México; 4 Laboratory of Molecular Genetics. Universidad Autónoma de Chiapas, Chiapas, México

## Abstract

**Background:**

Dengue viruses (DENV) attach to the host cell surface and subsequently enter the cell by receptor-mediated endocytosis. Several primary and low affinity co-receptors for this flavivirus have been identified. However, the presence of these binding molecules on the cell surface does not necessarily render the cell susceptible to infection. Determination of which of them serve as *bona fide *receptors for this virus in the vector may be relevant to treating DENV infection and in designing control strategies.

**Results:**

(1) Overlay protein binding assay showed two proteins with molecular masses of 80 and 67 kDa (R80 and R67). (2) Specific antibodies against these two proteins inhibited cell binding and infection. (3) Both proteins were bound by all four serotypes of dengue virus. (4) R80 and R67 were purified by affinity chromatography from *Ae. aegypti *mosquito midguts and from *Ae albopictus *C6/36 cells. (5) In addition, a protein with molecular mass of 57 kDa was purified by affinity chromatography from the midgut extracts. (6) R80 and R67 from radiolabeled surface membrane proteins of C6/36 cells were immunoprecipitated by antibodies against *Ae. aegypti *midgut.

**Conclusion:**

Our results strongly suggest that R67 and R80 are receptors for the four serotypes of dengue virus in the midgut cells of *Ae. aegypti *and in C6/36 *Ae*. *albopictus *cells.

## Background

Dengue (DEN) is distributed worldwide in tropical and subtropical countries including Mexico and the USA and is the most common vector-borne viral disease in humans. Infection ranges from asymptomatic or mild self-limited illness (dengue fever, DF) to a severe disease with spontaneous hemorrhaging (dengue hemorrhagic fever, DHF), or, most seriously, to DEN shock syndrome (DSS) characterized by circulatory failure. Fifty million DEN infections with 500,000 cases of DHF and 12,000 deaths occur each year [1]. In the years 2002 to 2004, 23,826 cases of DEN and 5,557 of DHF were reported in Mexico [2].

The Dengue virus (family *Flaviviridae*, genus *Flavivirus*, species *Dengue virus*) infects mammalian [3] and vector cells. Neutralizing antibodies facilitate the binding and penetration of DENV into human macrophages; the virus-antibody complex binds Fc receptors [4]. This mechanism does not explain virus entry in primary infections or in cells with non-Fc receptors. Transmission electron microscopy shows that DEN viruses must attach to the cells [5,6], suggesting that the cells must have virus receptor(s) on their surfaces to be susceptible to infection. Proteins [7-12], heparan sulfates [13], LPS/CD14-associated binding proteins [14] and other glycoproteins [15] have been proposed as cellular receptors for DENV. In addition, DC-SIGN [16-18] has been suggested as a mediator of DENV infection in dendritic cells and could participate in binding large numbers of other viruses such as HIV-1, Ebola and CytoMV to host cell surfaces [19].

In C6/36 cells, two major polypeptides have been described with apparent molecular weights of 67 and 80 kDa, which bind to DEN virus serotype 2 (DENV2) [9]. The mechanism by which the virus enters mosquito cells is unknown. The presence of receptor(s) on the surface does not necessarily render a cell susceptible to infection. In natural infection, DENV is first deposited in the mosquito vector and then in a human host bitten by the vector during a blood meal. Therefore, it is necessary to study receptors in the mosquito midgut (MG) to determine which binding proteins serve as true virus receptors; they may be relevant to treating DEN infection and in designing control strategies. This is especially important because mosquitoes differ in their susceptibilities to infection, resulting in different vector competences [20-22].

The present study shows that the proteins R80 and R67 are the putative receptors in the MG of *Ae. aegypti*. Moreover, proteins with the same apparent molecular weights were purified by DENV2 affinity chromatography from the MG of this mosquito and from C6/36 cells. In addition, a protein with molecular weight of 57 kDa was also purified from mosquito MG extracts by affinity chromatography. Immunoprecipitation of radiolabeled membranes using *Ae. aegypti *anti-MG antibodies showed that R80 and R67 are contained in mosquito cell membranes. Furthermore, the four serotypes of DENV recognized the same proteins on *Ae. aegypti *and C6/36 cells.

## Results

### Identification and purification of the putative DENV2 receptors in Ae. aegypti midgut by the virus overlay protein binding assay

**Figure 1:**
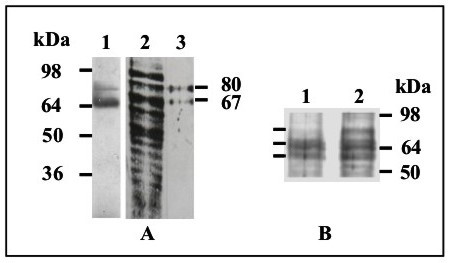
**VOPBA and DENV2 affinity chromatography of the virus dengue receptors**. Proteins from *Aedes aegypti *MGs were extracted with 0.05% Triton X-100, separated by 10% SDS-PAGE and blotted on to a PVDF membrane. The putative receptors were revealed after incubation with DEN2 virus and peroxidase-labeled goat anti-mouse antibody (panel A, lane 1) as described in the methods section. Receptors were purified from C6/36 cell membranes (panel A, lane 3) or MG (fractions 1 and 2, panel B) by DENV2 affinity chromatography using a DEN2-Sepharose™ 4B column as described in the methods section. The apparent molecular weights of these proteins are shown on the right side of panel A and on the left side of panel B. Proteins separated by SDS-PAGE were silver stained. The protein pattern of total C6/36 membranes is shown in panel A lane 2. Molecular weight markers are shown on the left side in panel A and on the right side in panel B.

Mosquito MG proteins were extracted with 0.05% of Triton X-100, separated by SDS-PAGE, blotted on to a PVDF membrane and incubated with biotinylated DENV2 virus to identify the putative receptors. Two prominent polypeptides with apparent molecular masses of 67 and 80 kDa were observed (Figure 1, panel A, lane 1). The negative control without virus showed no bands (data not shown).

To purify the putative receptors, DENV2 affinity chromatography was performed by passing cell membrane proteins from C6/36 cells from *Ae. albopictus *(Figure 1, panel A, lane 2) or MG proteins from *Ae. aegypti *through a DENV2-Sepharose™ 4B column. Representative patterns (from 8 experiments) of C6/36 cell membrane or midgut proteins retained and eluted by the column (EP) are shown in Figure 1 panel A, lane 3, and Figure 1 panel B, respectively. Two major proteins with molecular masses of 67 (R67) and 80 (R80) kDa were observed. Figure 1A lane 2 shows the total protein pattern of C6/36 cells. MG proteins eluted from the column showed an additional band with apparent molecular weight of 57 kDa (Figure 1, panel B). Fraction 2 showed the 80 kDa protein (Figure 1, panel B, lane 2).

### Immunoprecipitation of the putative receptors from C6/36 cell apical surfaces

**Figure 2:**
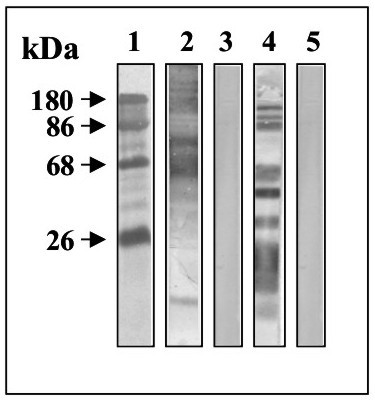
**Immunoblots of C6/36 total extracts**. Extracts of C6/36 cells (lanes 2, 3) or MG (lanes 4, 5) were subjected to 10% SDS-PAGE, blotted on to nitrocellulose and probed with anti-C6/36 membrane proteins (lane 2), pre-immune serum (lane 3), anti-MG (lane 4) or pre-immune serum (lane 5).

To confirm that the putative receptors in *Ae. aegypti *MG epithelial cells are similar to those in *Ae. albopictus *C6/36 cells, the apical surfaces of C6/36 cells were labeled with ^125^I and precipitated with anti-C6/36 cell membrane and anti-MG antibodies. Antibody specificity was tested by immunoblotting (Figure 2, panel A, lanes 2, 4). Proteins with apparent molecular weights over 180 kDa were recognized. The negative control with pre-immune serum showed no bands (Figure 2, lanes 3 and 5).

**Figure 3:**
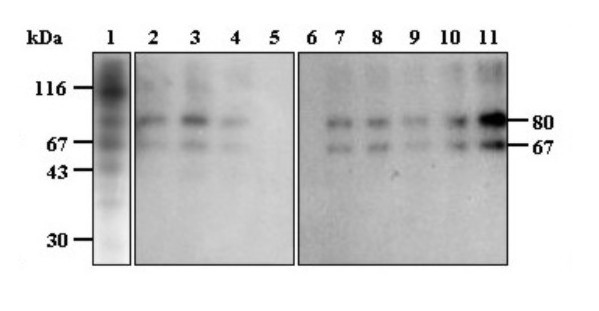
**Immunoprecipitation of labeled membrane proteins**. Proteins from C6/36 cell apical surfaces radiolabeled with ^125^I (lane 1) were immunoprecipitated with polyclonal anti-MG (lane 2) or anti-membrane (lane 3 and 4) and separated by 10% SDS-PAGE. The molecular weights of the immunoprecipitated proteins are shown on the left side. A negative control using an unrelated antibody (anti-actin) is shown in lane 5. Labeled apical cell surface proteins recognized by DEN1 (lane 7), DEN2 (lane 8), DEN3 (lane 9) and DEN4 (lane 10) were immunoprecipitated with specific anti-DENV as described in the methods section. A control without DEN virus was included (lane 6). ^125^I-labeled proteins immunoprecipitated by anti-EP were used as a positive control (lane 11). All proteins were separated by 10% SDS-PAGE and labeled proteins were detected by autoradiography. The molecular weights of these proteins are shown on the right side. Molecular weight markers are shown on the left side.

Labeled proteins from the apical surfaces of C6/36 cells (Figure 3, lane 1) precipitated by polyclonal antibodies against *Ae. aegypti *MG (Figure 3, lane 2) or C6/36 cell membranes (Figure 3, lane 3–4) were separated by 10% SDS-PAGE. The immunoprecipitated labeled proteins had molecular masses of 80 and 67 kDa. The labeled proteins were not precipitated by an unrelated antibody, anti-actin (Figure 3, lane 5).

### Apical cell surface proteins bound the four serotypes of DEN virus

To determine whether different serotypes of DENV recognized the same receptors, labeled apical cell surface proteins from C6/36 cells were incubated with DENV1, DENV2, DENV3 or DENV4 (Figure 3 lanes 7–10, respectively) and precipitated by incubation with antibodies against each DENV serotype. All four serotypes led to the immunoprecipitation of proteins with molecular masses of 80 and 67 kDa (Figure 3, lanes 7–10). Labeled C6/36 apical membrane proteins were precipitated by anti-EP antibodies as a positive control (Figure 3, lane 11). The control without DEN virus showed no polypeptides (Figure 3, lane 6).

### Polyclonal antibody-mediated blocking of DENV2 binding and infection of C6/36 cells

**Figure 4:**
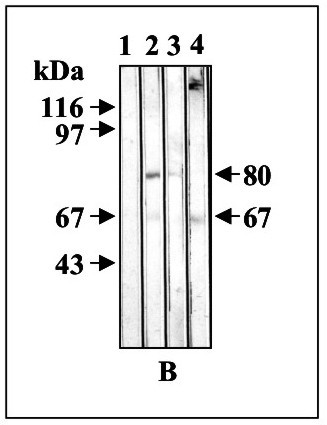
**Immunoblotting of total C6/36 extracts**. Extracts from C6/36 cells treated as in Figure 2 were tested with pre-immune mouse serum (lane 1), anti-EP (lane 2), anti-R80 (lane 3) or anti-R67 (lane 4). Molecular weight markers are shown on the left side and the proteins detected by the antibodies on the right side.

**Figure 5:**
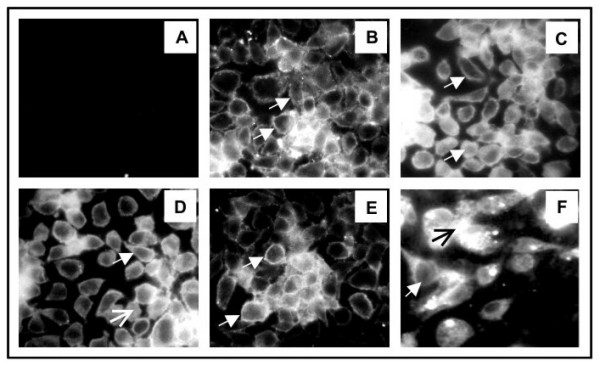
**Immunofluorescence microscopy of C6/36 cells**. Cell monolayers were stained with pre-immune serum (A), anti-cell membrane (B), anti-MG (C), anti-EP (D), anti-R67 (E) and anti-R80 (F) and then incubated with fluorescein-conjugated goat anti-mouse antibodies.

The specificities of anti-EP, anti-R80 and anti-R67 prepared to test inhibition of DEN virus binding and infection were assessed by immunoblotting (Figure 4) and immunofluorescence (Figure 5) as described in the methods section. Anti-EP recognized two polypeptides with molecular masses of 80 and 67 kDa (Figure 4, lane 2) and anti-R80 and anti-R67 recognized polypeptides of 80 (Figure 4, lane 3) and 67 kDa (Figure 4, lane 4), respectively. Pre-immune mouse serum recognized no polypeptides (Figure 4, lane 1).

There was no immunofluorescence staining when C6/36 cells were incubated with pre-immune serum (Figure 5A). In contrast, anti-membrane, anti-MG, anti-EP, anti-R67 and anti-R80 antibodies all stained the C6/36 membranes (Figure 5B–F, respectively). Anti-R80 also stained the cytoplasm (Figure 5F).

**Figure 6:**
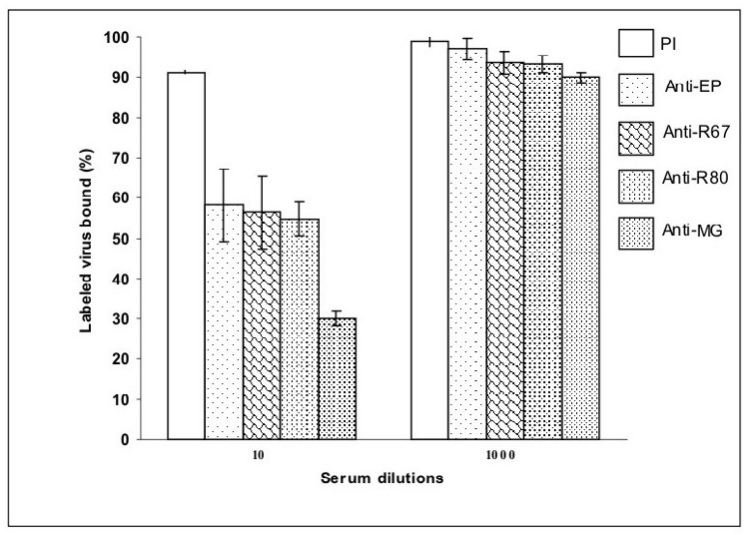
**Blocking of DEN-2 virus binding by specific antibodies**. C6/36 cell monolayers were incubated with 1:10 or 1:1000 diluted pre-immune serum (PI), anti-EP, anti-R67, anti-R80 or anti-MG. To assay the binding of radiolabeled virus, cells were dissolved in 3% SDS in all experiments. The total [^125^I]DENV2 virus bound was 5,286 ± 300 cpm. The experiments were repeated at least 3 times in quadruplicate.

In order to test antibody blocking of DENV binding, C6/36 cell monolayers were incubated with anti-EP, anti-R67, anti-R80 or anti-MG antibodies or pre-immune serum, then with ^125^I- DEN2 virus as described in the methods section (Figure 6). Anti-EP, anti-R67 and anti-R80 decreased virus binding by up to 40%. Binding was inhibited by 70% when anti-MG was diluted 1:10 (Figure 6).

**Figure 7:**
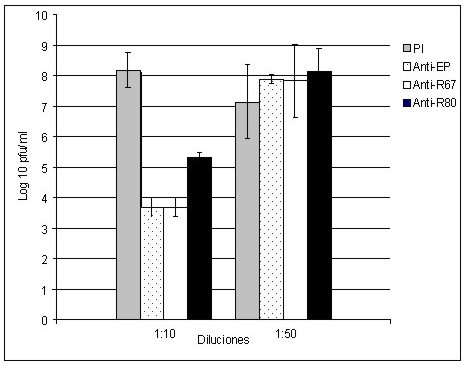
**Inhibition of DENV2 infection by specific antibodies**. C6/36 cell monolayers were incubated separately in the presence of pre-immune serum, anti-EP, anti-R67 or anti-R80 at 28°C for 60 min and then DENV serotype 2 was added (600 PFU/well) and incubated for 30 min at 4°C. After washing, fresh culture medium was added and the cells were incubated for 8 days. Pre-immune serum and antibodies were diluted 1:10 and 1:50. Viral titers in the supernatants were determined by viral plaque assay in LLC-MK2 cells. The experiments were repeated at least 3 times in quadruplicate.

To test the inhibition of DENV2 infection by the specific anti-receptor antibodies, C6/36 cell monolayers were incubated separately for 1 h in media containing pre-immune serum, anti-EP, anti-R67 or anti-R80 diluted 1:10 or 1:50, before being infected with DENV2. Infection was allowed to proceed for 8 days and the amount of infectious virus was determined by viral plaque assays in LLC-MK2 cells (Figure 7). Maximal inhibition of infection was obtained with anti-EP and anti-R67 diluted 1:10 (Figure 7). These antibodies inhibited DEN 2 replication in the C6/36 cells nearly 1000-fold. Anti-R80 showed a relatively minor inhibitory effect of approximately 100-fold. Anti-EP, anti-R67 or anti-R80 diluted 1:50, and pre-immune serum, did not inhibit DEN2 replication in these cells.

## Discussion

When a mosquito takes a viremic blood meal, virions interact with receptors on the midgut epithelial cells and penetrate and infect them. Arbovirus blocked at early stages of midgut infection is considered a midgut infection barrier (MIB). A midgut escape barrier (MEB) is considered when infectious virions do not disseminate to hemoceles, or disseminate but do not infect secondary target organs.

Most studies of flavivirus vector competence in *Ae. aegypti *indicate that MIB is a major determinant of transmission [23-25] and shows wide variation both among and within *Ae. aegypti *populations for flaviviruses including DENV [20,22,25]. Moreover, *Ae. aegypti *MG is generally considered the best candidate tissue for disrupting the virus life cycle within the mosquito because it is the earliest interface between insect and virus (see above). This strongly suggests that DENV attachment to MG epithelial cell receptors is critical for understanding the initial virus-vector interactions and will help to explain MIBs to DENV infection and variations in vector competence. Furthermore, we would expect that virus serotype and genotype would influence virus attachment to midgut receptors.

Thus, identification of viral receptors in the MG represents a critical step in understanding vector competence and designing possible targets for preventing viral entry to cells and therefore inhibiting the infection. Feasible approaches to intervention are monoclonal antibodies blocking the receptor, and synthetic peptides mimicking the viral receptor and thus competing with the host receptor for virus attachment. This will allow novel strategies for the control and prevention of DEN to be developed. Published data have shown that the viral E protein is involved in target cell recognition [6,26,27]. Recent structural and genetic evidence [28,29] suggests that the prM/M stem-anchor region is also likely to play a role in virion entry to cells. Purification by DENV 2 affinity chromatography using native virus is a novel approach to obtaining the putative protein receptors for DENV.

Several authors using the overlay assay have identified polypeptides with molecular masses of 27, 45, 67 and 87 in macrophages [8], 40 and 70 in myelomonocytes [30], 45 and 72 kDa in B and T cells from humans [31], and two heparan sulfate containing cell-surface binding proteins resolving at 19 and 37 kDa in hepatocytes [32]. The data in the present paper strongly support the view that the 67 and 80 kDa proteins are receptors for DENV 1, 2, 3 and 4 in MG cells from *Aedes aegypti*. Furthermore, we have purified these proteins for the first time from both *Ae. albopictus *C6/36 cells and *Ae. aegypti *MGs using DENV2 affinity chromatography; we also generated specific antibodies against them, which inhibited binding and infection of cultured cells by DENV 2. These results contrast with those of Yazi-Mendoza et al. [33], who showed that DENV 4 binds to a 45 kDa protein in mosquito tissues. The results in Figure 2 clearly demonstrate that DENV 4 recognizes R67 and R80 in C6/36 cell membranes. A plausible explanation is that Yazi-Mendoza et al. [33] used total cell extracts for their VOPBA, while our results were obtained from plasma membranes, although viruses are known to have several receptors [7]. In any event, further experiments are needed to settle the point. Interestingly, Jindadamrongwech & Smith [7] found a serotype-specific heterogeneity among DENV binding proteins on HepG2 human Liver cells. It is therefore possible that different cell lines have different specificities for virus serotypes.

The specific antibodies against R67 and R80 inhibited radiolabeled DEN virus binding and the highest inhibition was observed with anti-MG antibodies, suggesting that other cell membrane molecules may participate in virus binding. Candidates include non-sialic acid carbohydrates, since we previously showed that sialic acid does not participate in virus binding [9]. In agreement with our results, Zieler et al. [29] failed to detect sialic acid in *Ae. aegypti *MG. More recently, Thaisomboonsuk et al. [34] reported the inability of neuroaminidase to inhibit DEN-2 virus binding to insect cells.

As we mentioned earlier, DC-SIGN has also been reported as a functional receptor on human dendritic cells [15,16] for DENV and other flaviviruses. Further studies are needed to determine the involvement of DC-SIGN as receptor in DENV infection in *Ae. aegypti *mosquitoes.

Collectively, the results corroborate the notion that DENV utilizes multiple cell surface molecules for binding to and infection of target cells, some of which may be common to all cells and shared among several viruses [6]. Interestingly, Sakoonwtanyoo et al. [12] suggested that one of these protein receptors for DENV 2, 3 and 4 may be a laminin-binding protein with an apparent mass of 50 kDa. Although we failed to detect a protein with this molecular mass, this does not eliminate the possibility.

In summary, specific membrane molecules are required for DENV binding and the nature of these molecules seems to depend on cell type. Interestingly, our results show that R67 and R80 are the putative receptors in the MG of *Ae. aegypti *mosquitoes, the principal DENV vector in America, and in *Ae. albopictus *C6/36 cells. In addition, these receptors are specific for all four serotypes of DENV.

## Conclusion

This paper documents for the first time that the proteins R67 and R80 are putative receptors for dengue virus in the midgut of *Ae. aegypti *and in *Ae. albopictus *C6/36 cells, since specific antibodies against these proteins inhibited the binding and cytopathic effects of the virus. Furthermore, we have shown that these receptors are specific for all four serotypes of DENV.

## Methods

### Mosquito culture

*A. aegypti *mosquitoes collected as larvae in Monterrey, Mexico were laboratory-reared and maintained at 28 ± 2°C and 80% RH under a 12:12 h (L:D) photoperiod using standard mosquito-rearing procedures [35]. After day 4, adult MGs were dissected and stored at -80°C until use.

### DENV infection cells

*Ae. albopictus *clone C6/36 cells were grown at 28°C as previously described [36]. After 18 h of culture, the cells (2 × 10^6^/100 mm plate) were infected with 0.2 ml DENV2 inoculum with an input MOI of 600 PFU per plate and incubated at 28°C for 10 days.

### Virus purification

The flaviviruses used in this study were: DENV1, strain Hawaii; DENV2, strain New Guinea C (NGC); DENV3, strain H-87; and DENV4, strain H-341. They were obtained from Dr. D. J. Gubler (Division of Vector-borne Infectious Diseases, Centers for Disease Control, Fort Collins, CO, USA) and kindly provided by Dr. Blanca Ruiz (Biomedicas, UNAM).

Virus passage in Vero cells was clarified from cell culture supernatants. Viruses were concentrated and purified as described by Putnak et al. [37]. Titers of virus stocks made in LLC-MK2 cells [38] were 8 × 10^8 ^PFU/ml for each DENV strain. Control antigens harvested from uninfected Vero cells were prepared in the same manner.

The purified viruses were iodinated using 1 mCi of ^125^I (Amersham Pharmacia Biotech) as described elsewhere [39]. The specific activity was 4.7 × 10^9 ^cpm/mg of protein.

Biotinylation was performed as described previously [9]. The viral pellet was stored at -70°C until use.

### Membrane preparation

C6/36 cell membranes were prepared essentially as described elsewhere [40] by scraping the cells from confluent plates in the presence of PBS. This procedure has been described in detail for C6/36 cells [9]. Labeled membrane proteins were identified at the 20% interface of the gradient and verified by SDS-PAGE.

### DENV2 affinity chromatography

DEN2 viruses (8 × 10^8 ^PFU/ml) were bound covalently to 1 g of CNBr-activated Sepharose™ 4B as recommended by the manufacturer (Amersham Biosciences). The DENV2-Sepharose™ column was stored in 0.002% sodium azide at 4°C until use.

The protein extract was obtained by homogenizing MGs (300/ml) in buffer E (50 mM Tris-HCl, pH 7.2, 1 mM EDTA, 0.05% v/v Triton X-100) containing P 8340 protease inhibitor cocktail from Sigma. To obtain the soluble proteins, the homogenate was centrifuged for 10 min at 7245 × g at 4°C.

MG protein extract (2 mg), or 100 μg C6/36 cell membranes [4,9], was applied to a DENV2-Sepharose™ 4B column (1 ml) equilibrated in buffer E and washed with the same buffer. The DENV2-binding proteins were eluted with buffer E containing 0.5 M NaCl. Fractions of 0.5 ml were collected, and the protein content was monitored by the Bradford method [41] and analyzed by polyacrylamide gel electrophoresis in the presence of sodium dodecyl sulfate on 10% gels [42]. The eluted proteins (EP) were stored at -70°C.

### Radiolabeling of C6/36 cell membrane proteins

Proteins from the apical surfaces of confluent monolayers were iodinated by the lactoperoxidase method [39]. Labeled membrane proteins (specific activity 1.4 × 10^6^cpm/mg) in buffer E were used immediately or stored at -70°C.

### Antibodies

Monoclonal anti-DENV2 antibodies were obtained from murine hybridomas (HB-46) from ATCC. Anti-DENV1, DENV3 and DENV4 were kindly provided by Dr. Garry Clark from CDC-Puerto Rico.

To obtain specific anti-R80 and anti-R67, proteins retained by the DENV2-Sepharose™ 4B column were separated by 10% SDS-PAGE. After silver staining, each of the two main protein species (R80, R67) was excised from the gel, cut in small pieces, suspended in PBS and mixed with an equal volume of Titer-Max adjuvant (CyTRx Corporation) to immunize two groups of BALB/c mice. Pre-immune sera were obtained before immunization. Proteins eluted (EP) from the DENV2 affinity column were used to obtain specific polyclonal antibodies. A total of 10 μg of this protein was used to immunize the mice. MG extracts (100 μg), or C6/36 cell membrane extracts (50 μg), were also used to immunize mice [9]. After fifteen days, the mice received a booster; they were bled after thirty days. Sera were stored at -70°C until use. Antibody specificity was tested by ELISA and immunofluorescence. Negative controls using pre-immune sera were included in all assays.

### Binding and infection blocking assays

C6/36 cells (1.5 × 10^4^) were cultured overnight in 96-well plates as described above. For binding assays, the cells were incubated for 60 min at 4°C with pre-immune serum, anti-EP, anti-R80, anti-R67 or anti-MG diluted 1:10 or 1:1000 in serum-free medium. Other cells were left untreated. ^125^I-DENV of each serotype was added (600 PFU/well) and incubated for 30 min at 4°C. After washing to remove non-bound labeled virus, cells containing the bound virus were solubilized in 3% SDS and the radioactivity was counted. For infection assays, cells were incubated for 60 min with pre-immune serum, anti-EP, anti-R80 or anti-R67 diluted 1:10 or 1:50 at 28°C and then DENV serotype 2 was added (600 PFU/well) followed by incubation for 30 min at 4°C. After washing, fresh culture medium was added and the cells were incubated for 8 days. The viral titer was determined in each supernatant by viral plaque assay in LLC-MK2 cells [43]. Four wells were assessed for each experimental condition.

### Immunofluorescence

C6/36 cell monolayers were stained as previously described [9]. Pre-immune sera as well as each of the polyclonal antibodies (anti-R80, anti-R67 and anti-MG) were diluted 1:100 and incubated overnight at 4°C. After washing with PBS, the cells were incubated with fluorescein-conjugated goat anti-mouse (1:500) antibodies (Hyclone Laboratories, Inc., Utah). After further washing, they were mounted in 50% glycerol and observed by fluorescence microscopy.

### Electrophoretic blotting

Proteins were electrophoresed in 10% SDS-PAGE [42] and transferred to nitrocellulose paper [44]. Membranes were incubated with anti-EP, anti-R80, anti-R67 or anti-MG diluted 1:50. The goat anti/mouse IgG second antibody was conjugated to alkaline phosphatase and color development was measured as recommended by the manufacturer (Zymed Laboratories).

### Virus overlay protein binding assay (VOPBA)

Mosquito MG proteins were separated by 10% SDS-PAGE as described above and blotted on to PVDF membranes (BioRad) by Towbin's technique [44]. The procedure was followed as previously described [9] with some modifications. Briefly, the electrophoretic blots were incubated overnight with unlabeled virus, washed with PBS and incubated overnight with monoclonal anti-flavivirus (diluted 1:100) at 4°C. After washing with PBS, they were incubated for 2 h at room temperature with peroxidase-labeled goat anti-mouse (Kirkegaarde Perry Laboratories, USA) diluted 1:1000 in 5% skimmed milk in PBS. Finally, they were washed with PBS, and reactive proteins were visualized by developing with the chromogenic substrate 4-chloro-1-naphthol/hydrogen peroxide. The reaction was stopped after 1 h by washing with water.

### Receptor-virus immunoprecipitation

Radiolabeled cell membrane proteins (5 μg) solubilized in buffer E containing protease inhibitors (see above) were incubated for 2 h at 37°C with 10 μl of each DENV serotype (6.0 × 10^6 ^PFU/ml) and anti-DENV 1, 2, 3 or 4 antibodies diluted 1:100. The mixture was centrifuged at 5,000 × g for 15 min and washed twice with PBS. The supernatant was discarded and the proteins from the pellet were suspended in SDS gel-loading buffer and separated by 10% SDS-PAGE. Labeled polypeptides were detected by autoradiography [45]. Negative controls were included by incubating the same preparations with anti-actin, an unrelated antibody.

## Authors' contributions

RFMC carried out the virus purification experiments, DENV infection of the cells, affinity purification of DENV2 MG receptor, electrophoretic blotting and the virus overlay protein binding assay. HAEA purified the receptors from C6/36 cell membranes, and carried out the binding and infection blocking assays and immunofluorescence studies. RT radiolabeled the membrane proteins and obtained the membrane preparations, raised antibodies and performed the receptor-virus immunoprecipitation. ADB cultured and field collected mosquitoes. MCN assembled the manuscript and participated in data analysis. MLM proof-read and assembled the manuscript. All authors read and approved the final manuscript.
